# Loss of 24-hydroxylated catabolism increases calcitriol and fibroblast growth factor 23 and alters calcium and phosphate metabolism in fetal mice

**DOI:** 10.1093/jbmrpl/ziae012

**Published:** 2024-01-29

**Authors:** David Bennin, Sarah A Hartery, Beth J Kirby, Alexandre S Maekawa, René St-Arnaud, Christopher S Kovacs

**Affiliations:** Faculty of Medicine – Endocrinology, Memorial University of Newfoundland, St. John’s, Newfoundland and Labrador, A1B 3V6, Canada; Faculty of Medicine – Endocrinology, Memorial University of Newfoundland, St. John’s, Newfoundland and Labrador, A1B 3V6, Canada; Faculty of Medicine – Endocrinology, Memorial University of Newfoundland, St. John’s, Newfoundland and Labrador, A1B 3V6, Canada; Faculty of Medicine – Endocrinology, Memorial University of Newfoundland, St. John’s, Newfoundland and Labrador, A1B 3V6, Canada; Shriners Hospitals for Children–Canada and McGill University, Montréal, Quebec, H4A 0A9, Canada; Faculty of Medicine – Endocrinology, Memorial University of Newfoundland, St. John’s, Newfoundland and Labrador, A1B 3V6, Canada

**Keywords:** genetic animal models, PTH/Vitamin D/FGF23, disorders of calcium/phosphate metabolism, fetus, placenta, system biology – bone interactors

## Abstract

Calcitriol circulates at low levels in normal human and rodent fetuses, in part due to increased 24-hydroxylation of calcitriol and 25-hydroxyvitamin D by 24-hydroxylase (CYP24A1). Inactivating mutations of *CYP24A1* cause high postnatal levels of calcitriol and the human condition of infantile hypercalcemia type 1, but whether the fetus is disturbed by the loss of CYP24A1 is unknown. We hypothesized that loss of *Cyp24a1* in fetal mice will cause high calcitriol, hypercalcemia, and increased placental calcium transport. The *Cyp24a1^+/−^* mice were mated to create pregnancies with wildtype, *Cyp24a1^+/−^*, and *Cyp24a1* null fetuses. The null fetuses were hypercalcemic, modestly hypophosphatemic (compared to *Cyp24a1^+/−^* fetuses only), with 3.5-fold increased calcitriol, 4-fold increased fibroblast growth factor 23 (FGF23), and unchanged parathyroid hormone. The quantitative RT-PCR confirmed the absence of *Cyp24a1* and 2-fold increases in *S100g*, sodium–calcium exchanger type 1, and calcium-sensing receptor in null placentas but not in fetal kidneys; these changes predicted an increase in placental calcium transport. However, placental ^45^Ca and ^32^P transport were unchanged in null fetuses. Fetal ash weight and mineral content, placental weight, crown-rump length, and skeletal morphology did not differ among the genotypes. Serum procollagen 1 intact N-terminal propeptide and bone expression of sclerostin and *Blgap* were reduced while calcitonin receptor was increased in nulls. In conclusion, loss of *Cyp24a1* in fetal mice causes hypercalcemia, modest hypophosphatemia, and increased FGF23, but no alteration in skeletal development. Reduced incorporation of calcium into bone may contribute to the hypercalcemia without causing a detectable decrease in the skeletal mineral content. The results predict that human fetuses bearing homozygous or compound heterozygous inactivating mutations of *CYP24A1* will also be hypercalcemic in utero but with normal skeletal development.

## Graphical Abstract

**Graphical Abstract:**
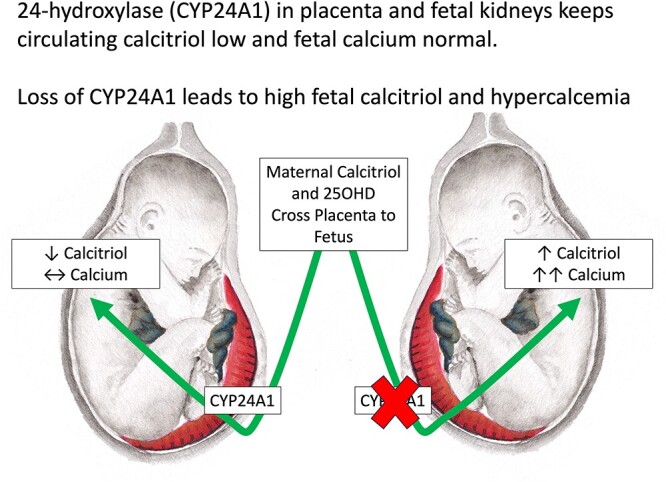

## Introduction

Calcitriol (the active or hormonal form of vitamin D) is critically important for the regulation of calcium and bone metabolism in children and adults. Its main function is to upregulate the active and passive intestinal absorption of calcium and phosphate, thereby supplying mineral to the skeleton.^1^ This role has been most clearly revealed through the study of the absence of calcitriol or of the vitamin D receptor (VDR). In human and animal models (reviewed in detail in study^2^), severe vitamin D deficiency, genetic inability to make calcitriol (loss of CYP27B1),^2–5^ or genetic inability to respond to calcitriol (inactive VDRs), each result in reduced intestinal calcium and phosphate absorption, hypocalcemia, hypophosphatemia, secondary hyperparathyroidism, and under-mineralized softened bone (rickets or osteomalacia).

What role does calcitriol play in fetal mineral metabolism and skeletal development? The VDRs are widely expressed in embryonic mesenchyme that becomes the skeletal tissues, in chondrocytes and osteoblasts within the growth plates of the fetal skeleton, and within placental cells that actively transport calcium and phosphate.^2^ Moreover, placental trophoblasts and fetal kidneys abundantly express CYP27B1 (1α-hydroxylase), which converts 25-hydroxyvitamin D (25OHD) into calcitriol and 24-hydroxylase (CYP24A1), which catabolizes 25OHD and calcitriol. These findings predict that calcitriol should have important roles in fetal mineral homeostasis and skeletal development.

However, calcitriol is not required to regulate fetal mineral metabolism, or for the development and mineralization of the fetal skeleton.^2^ Severely vitamin D-deficient pregnant rats bear pups with normal serum calcium, phosphorus, magnesium, skeletal morphology, and ash weight.^6–9^ Studies of *Vdr* null and *Cyp27b1* null fetal mice,^10–12^ and *Cyp27b1* null Hannover pigs,^13^ have shown that the absence of either VDR or calcitriol does not affect fetal serum calcium, phosphorus, magnesium, and parathyroid hormone (PTH); amniotic fluid mineral content (a surrogate of renal mineral excretion); and skeletal morphology, mineral content, and gene expression. Placental calcium transfer was normal in vitamin D-deficient rat fetuses,^9^*Cyp27b1* null fetal mice,^12^ and *Cyp27b1* null fetal Hannover pigs.^13^ Conversely, placental calcium transport was increased in *Vdr* null mice, which had high serum calcitriol concentrations that presumably acted on an alternate receptor to stimulate transport.^10^^,^^11^

Human data have also shown that vitamin D and calcitriol are not required for fetal mineral homeostasis and skeletal development. Observational studies comparing vitamin D-replete to -deficient human pregnancies, and randomized clinical trials of vitamin D supplementation during pregnancy, have found no effect of vitamin D deficiency on cord blood calcium, phosphorus, or PTH; radiological evidence of rickets; or (in babies dead from obstetrical accidents) skeletal ash weight or calcium and phosphorus content of skeletal ash.^2^^,^^14–16^ Babies with 1α-hydroxylase deficiency (pseudovitamin D deficiency rickets or vitamin D-dependent rickets type I) and those lacking VDRs (hereditary vitamin D-resistant rickets or vitamin D-dependent rickets type II) are normal at birth with normal serum calcium^17^^,^^18^; it is generally not until infancy or the second yr of life that hypocalcemia, hypophosphatemia, and rickets develop in these children.^4^^,^^17^ Overall, these clinical findings suggest that calcitriol plays no essential role during human fetal development with respect to calcium, phosphorus, and skeletal homeostasis. This is likely because the placenta is supplying mineral to the developing fetus rather than the intestines, and the placenta does not require calcitriol-mediated pathways to transport mineral. It is after birth, when intestinal calcium and phosphorus absorption are reduced by the inadequate actions of calcitriol, that mineral homeostasis and skeletal mineralization become impaired.

The fetal circulation is characterized by low circulating levels of calcitriol, due to active 24-hydroxylation of calcitriol and 25OHD, and the suppressive effects of high serum calcium and phosphorus, low PTH, and fibroblast growth factor 23 (FGF23).^2^^,^^14^ Catabolism by CYP24A1 results in 40-fold higher concentrations of 24,25(OH)_2_D as compared to calcitriol in human cord blood^19–21^ and in fetal rodents and lambs.^22–24^ In our prior studies of fetal mice, 24,25(OH)_2_D was 54-fold higher than calcitriol and 3 times higher than maternal 24,25(OH)_2_D.^12^ Postnatally, high CYP24A1 activity in neonatal rodents and lambs continues to catabolize 25OHD and contributes to low levels of calcitriol.^25^^,^^26^ Collectively, these studies suggest that developmental programming maintains low serum calcitriol until the intestines are ready to respond to calcitriol after birth.

Absence of *Cyp24a1* in mice causes postnatal lethality in about 50% of neonates due to severe hypercalcemia (twice the wildtype [WT] value), which is accompanied by a marked elevation in calcitriol and undetectable PTH.^27^^,^^28^ These mutant mice model the human condition of infantile hypercalcemia type 1, which is caused by homozygous and compound heterozygous inactivating mutations of *CYP24A1.*^29–31^ Affected human neonates have hypercalcemia, elevated calcitriol, suppressed PTH, reduced CYP24A1 activity, and reduced 24-hydroxylated metabolites in the circulation. Other affected individuals remain undetected unless mild hypercalcemia, hypercalciuria, nephrocalcinosis, and nephrolithiasis are discovered when they become older children or adults.^31^ Conversely, affected women have presented during pregnancy with severe and even life-threatening hypercalcemia.^32–34^ This is likely due to the normal pregnancy-related rise in calcitriol being much higher due to being unopposed by catabolism, and thereby, leading to a markedly increased intestinal calcium absorption.

The effect that loss of CYP24A1-mediated catabolism has on fetal mineral metabolism and skeletal development is unknown. It is likely that human data will never be available due to delayed recognition of the condition (months to years). In prior studies, *Cyp24a1* null mice were examined beginning 5 d after birth.^27^

In the present study, we hypothesized that *Cyp24a1* ablation would cause increased serum calcitriol, hypercalcemia, and altered placental mineral transport. These studies should inform whether the human condition of severe infantile hypercalcemia will be preceded by disturbances in the cord blood minerals as well as calciotropic and phosphotropic hormones.

## Materials and methods

### Animal husbandry and experimental time points

The *Cyp24a1* null mice (a global knockout) were generated in the laboratory of Dr. René St-Arnaud.^27^^,^^28^ The colony has been maintained in a pathogen-free facility by mating *Cyp24a1^+/−^* mice together and periodic backcrossing into the parent C57BL/6 strain. Experimental mice were generated by mating male and female *Cyp24a1^+/−^* mice to create pregnancies bearing WT, *Cyp24a1^+/−^*, and *Cyp24a1* null fetuses. Additional controls for assessment of serum calcium and phosphorus were generated by mating related WT females to the same *Cyp24a1^+/−^* males, thereby bearing WT and *Cyp24a1^+/−^* fetuses.

Male mice were housed singly, while females were housed up to 4 per cage. After the confirmation of pregnancy, the females were kept 1–2 per cage. The mice consumed a standard chow (2018 18% Protein Teklad Global Rodent Diet, Envigo, Madison, WI).

Mice of 10–12 wk of age were mated overnight; the presence of a vaginal mucus plug on the morning after mating marked the embryonic day (ED) 0.5. Females were kept singly in cages once confirmed to be pregnant. Normal gestation is 19 d, and fetal analyses were done at ED 18.5, except for ED 17.5, for measurement of placental mineral transport and collection of amniotic fluid.

The Institutional Animal Care Committee of Memorial University of Newfoundland approved all procedures involving live animals.

### Chemical and hormone assays

Sera and amniotic fluid were collected using methods previously described.^35^^,^^36^ Amniotic fluid was obtained from fetuses collected on ED 17.5 because it is too scant and viscous on ED 18.5. Calcium, phosphorus, and magnesium were analyzed using colorimetric assays (Sekisui Diagnostics PEI Inc., Charlottetown, PEI). Enzyme-linked immunosorbent assays were used to measure intact PTH (Immutopics, San Clemente, CA), calcitriol, procollagen 1 intact N-terminal propeptide (P1NP), and C-telopeptide (CTX) (Immunodiagnostic Systems Ltd., Boldon, Tyne and Wear, United Kingdom), and intact FGF23 (Kainos, Japan). Any raw values below an assay’s sensitivity were reset to values that equaled its detection limit, whereas raw values exceeding the upper limit were reset to equal the upper limit.

### Placental calcium and phosphate transfer

In brief, on ED 17.5, pregnant dams were given an intracardiac injection of 0.74 MBq ^45^Ca or ^32^P together with 0.74 MBq ^51^Cr-EDTA (EDTA is passively transferred and serves as a blood diffusional marker).^36^ Five minutes later, the fetuses were removed and later solubilized. The ^45^Ca or ^32^P activity within each fetus was measured using a liquid scintillation counter, while the ^51^Cr activity was assayed with a γ-counter. Each fetal ^45^Ca/^51^Cr or ^32^P/^51^Cr value was normalized within its litter to the mean heterozygous value in order that the results from different litters could be analyzed together. The ^45^Ca and ^32^P activities were also separately analyzed after normalizing to the mean heterozygous value within each litter.

### Fetal ash and skeletal mineral assay

As previously described,^37^ intact fetuses (ED 18.5) were weighed, crown-rump length measured, and then reduced to ash in a furnace (500 °C × 24 hr). A Perkin Elmer 2380 Atomic Absorption Flame Spectrophotometer assayed the calcium and magnesium content of the ash, while the phosphorus content was determined using a colorimetric assay (Sekisui Diagnostics).

### Alizarin red S and Alcian blue skeletal preparations

As previously described,^37^ skin, viscera, and adipose tissue were carefully removed from the fresh fetuses (ED 18.5). Each fetus was fixed in 95% EtOH for 5 d, followed by acetone for 2 d, to remove remaining fat and firm up the specimen. They were stained for 3 d in 10 mL of freshly prepared staining solution at 37 °C (1 volume 0.3% Alcian blue 8GS in 70% EtOH, 1 volume 0.1% Alizarin red S in 95% EtOH: 1 volume acetic acid: 17 volumes 70% EtOH). Then, they were washed in distilled water, followed by immersion in 1% aqueous KOH until the fetal skeleton was clearly visible (approximately 12–48 hr). They were cleared in 1% KOH containing increasing concentrations (20%, 50%, and 80%) of glycerine (7–10 d at each step). Finally, they were transferred into 100% glycerine for permanent storage.

### Histology

Undecalcified fetal tibias were fixed in 10% buffered formalin, dehydrated in graded alcohol series, embedded in paraffin, and stained with 2% methyl green. von Kossa staining was performed on 5-μm deparaffinized sections using 3% aqueous silver nitrate solution and 45 min of exposure to bright light.

### RNA extraction and real-time qPCR

Placentas, fetal kidneys, and hind limbs were snap-frozen in liquid nitrogen. Total RNA was purified using the RNeasy Midi Lipid kit for placentas and the Mini Lipid kit for fetal kidneys and hind limbs (Qiagen, Toronto, ON). The RNA quantity and quality were confirmed with the Agilent 2100 BioAnalyzer (Agilent Technologies, Santa Clara, CA). TaqMan Gene Expression Assays (with the manufacturer’s pre-designed primers and probes for optimal amplification; see Supplementary Table S1), and Fast Advanced Master Mix from Applied BioSystems (ABI)/Life Technologies (Burlington, ON), were used to determine the expressions of *Cyp24a1*, *Cyp27b1,* Ca^2+^-ATPase (*Pmca1*), calbindin-D9k (*S100g*), transient receptor potential vanilloid 6 (*Trpv6*)*, Pth*, PTH-related protein (*Pthrp* or *Pthlh*), *Vdr,* sodium–calcium exchanger type 1 *(Ncx1)*, the sodium–phosphate transporters *NaPi2a*, *NaPi2b*, and *NaPi2c,* phosphate transports *Pit1*, *Pit2*, *and Xpr1*, calcium-sensing receptor (*Casr*), protein disulfide isomerase family A, member 3 *(Pdia3),* sclerostin (*Sost*)*,* osteocalcin (*Bglap*), bone sialoprotein (*Ibsp*), alkaline phosphatase (*Alp1*), PTH/PTHrP receptor (*Pthr1*), *Runx2, Rank,* calcitonin receptor (*Calcr*), cathepsin K (*Ctsk*), and acid phosphatase (*Acp5*). The reference gene used for these studies was *Gapdh*. Details of conditions and cycle times have been previously reported.^38–40^ Briefly, cDNA was synthesized using the Taqman High Capacity cDNA Reverse Transcription Kit (ABI), and multiplex quantitative RT-PCR (qPCR) reactions were run in triplicate on the ViiA 7 Real-Time PCR System (ABI).^10^^,^^39^ The minimum sample size was 5 for each genotype. Relative expression was determined from the threshold cycle (*C_T_*) normalized to the reference gene.

### Statistical analyses

Data were analyzed using StatPlus:Mac Pro, Build 8.0.4.0 (AnalystSoft Inc, Vancouver, BC). ANOVA was used for analysis of biochemical, transport, and ash data, with Tukey’s post hoc test used to determine which pairs of means differed significantly. Where all 3 genotypes were included, the primary analysis used *Cyp24a1^+/−^* as the more stable comparator since these represented 50% of the fetuses in a litter. The qPCR data were analyzed by the Comparative C_T_ Method (2^Δ*CT*^).^41^ Two-tailed probabilities for all data are reported as mean ± SD.

## Results

No intrauterine demise of *Cyp24a1* null fetuses occurred, as a sampling of consecutive litters revealed genotypes in the following ratios: 101 WT, 226 *Cyp24a1^+/−^*, and 116 *Cyp24a1* null, or 1:2:1.

**Figure 1:**
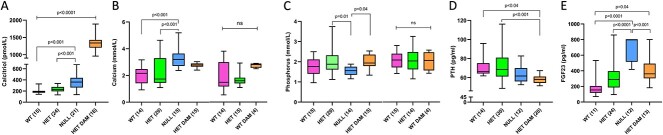
Biochemical and hormonal data from 24-hydroxylase (*Cyp24a1*) null fetuses and their littermates. The *Cyp24a1* null fetuses had 3.5-fold higher serum calcitriol that remained below the high maternal value of pregnancy (A). Serum calcium was significantly elevated in *Cyp24a1* null fetuses but not in their littermates (B, left graph), while serum calcium of WT and *Cyp24a1^+/−^* fetuses was unaffected by the dam being *Cyp24a1^+/−^* or WT (compare left to right graphs in B). Serum phosphorus was modestly but significantly reduced in *Cyp24a1* null fetuses (C, left graph), with serum phosphorus of WT and *Cyp24a1^+/−^* fetuses unaffected by a *Cyp24a1^+/−^* or WT dam (compare left to right graphs in C). Serum PTH did not differ across the fetal genotypes (D), while FGF23 was increased 4-fold in *Cyp24a1* null fetuses and exceeded the maternal value (E). The increase in FGF23 is underestimated due to 8 null values being increased above the upper limit of normal but reset to that upper limit. The numbers of observations are indicated in parentheses.

Serum calcitriol was increased 2-fold in *Cyp24a1* null fetuses as compared to their WT and *Cyp24a1^+/−^* littermates, although it remained well below the high maternal level achieved during pregnancy (Figure 1A). Increased calcitriol was accompanied in *Cyp24a1* nulls by hypercalcemia (Figure 1B, left-hand graph) and hypophosphatemia (Figure 1C, left-hand graph; compared to *Cyp24a1^+/−^* only). By comparison, serum calcium and phosphorus were no different between WT and *Cyp24a1^+/−^* fetuses and were unaltered by whether the dam was WT or *Cyp24a1^+/−^* (compare left- and right-hand paired graphs within each of Figure 1B and C). Maternal serum calcium and phosphorus were also no different between WT and *Cyp24a1^+/−^* dams (compared dam values within left- and right-hand paired graphs within each of Figure 1B and C).

PTH was not significantly altered in *Cyp24a1* null fetuses (Figure 1D), while FGF23 in *Cyp24a1* nulls was increased 3.5-fold over WT values and exceeded the maternal value (Figure 1E). Eight of the FGF23 values in *Cyp24a1* nulls and 1 value in *Cyp24a1^+/−^* fetuses exceeded the upper limit of the assay (800 pg/mL) and were reset to that value; consequently, the true FGF23 value was likely much higher in *Cyp24a1* nulls.

**Figure 2:**
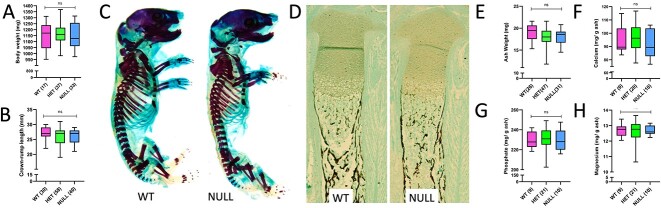
Skeletal parameters of 24-hydroxylase (*Cyp24a1*) null fetuses. Wet body weight (A) and crown-rump length (B) were unchanged in *Cyp24a1* null fetuses. In (C), representative images of fetal skeletons (ED 18.5) stained with alizarin red (for mineral) and Alcian blue (for cartilage) show that skeletal morphology of the *Cyp24a1* null was consistently normal, as shown by the normal crown-rump length, lengths of long bones, and mineralization pattern. In (D), representative sections of fetal tibias (ED 18.5) also show the normal morphology of the tibias, the lengths of the growth plates and bone shaft, periosteal thickness, and mineral (black) in the shafts of the tibias. The *Cyp24a1* null fetuses also had normal ash weight (E) and ash content of calcium (F), phosphorus (G), and magnesium (H). The numbers of observations are indicated in parentheses. Scale bars indicate 0.5 cm in (C) and 100 μm in (D).

The *Cyp24a1* null body weight (Figure 2A) and crown-rump lengths (Figure 2B) were no different than their WT littermates. To control for the variability in fetal size from litter to litter (larger litters mean smaller fetuses), we also adjusted for the mean values of *Cyp24a1^+/−^* pups within each litter, and there remained no differences in the body weight or crown-rump length (Supplementary Figure S1A and B). Examination of intact, stained skeletons demonstrated no change in the overall skeletal morphology of the *Cyp24a1* nulls (Figure 2C), and at the microscopic level, there was no change in lengths, cartilaginous versus bone compartments, or mineralization pattern of the tibias (Figure 2D). Skeletal ash weight (Figure 2E) and ash mineral content (Figure 2F–H) were also unaltered in *Cyp24a1* nulls.

**Figure 3:**
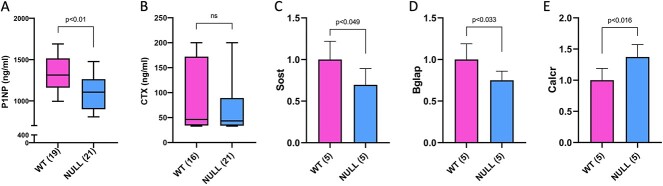
Biochemical markers of bone turnover and expression of bone-relevant genes. Serum procollagen 1 intact N-terminal propeptide was reduced in 24-hydroxylase (*Cyp24a1*) null compared to WT fetuses (A), while serum C-telopeptide was unchanged (B). The osteoblast-specific genes sclerostin (C) and osteocalcin (D) were reduced in *Cyp24a1* null hind limb bones, while expression of the osteoclast-specific gene calcitonin receptor (*Calcr*) was increased (E). Additional genes relevant to osteoblasts and osteoclasts showed no changes in expression; these are shown in Table 1. The numbers of observations are indicated in parentheses.

Assessment of biomarkers of bone turnover revealed reduced P1NP and normal CTX in *Cyp24a1* nulls (Figure 3A and B). Analysis of the expression of genes relevant to osteoblast differentiation and function in the hindlimb showed reduced *Sost* and *Bglap* in *Cyp24a1* null fetuses with no changes in the remaining genes (Figure 3C and D and Table 1), while among genes relevant to osteoclasts, *Calcr* was increased in *Cyp24a1* null fetuses (Figure 3E) and the others showed no changes (Table 1).

**Table 1:** Relative expression of genes from WT and *Cyp24a1* null hind limb bones. All values have been normalized to the WT; sample sizes were 5 per group. The results for *Sost*, *Bglap*, and *Calcr* are shown in Figure 3.

**Gene**	**WT**	***Cyp24a1* null**	***P*-value**
*Ibsp*	1.000 ± 0.180	0.835 ± 0.297	<.320
*Alp1*	1.000 ± 0.451	0.829 ± 0.202	<.470
*Pthr1*	1.000 ± 0.071	1.051 ± 0.311	<.797
*Runx2*	1.000 ± 0.240	0.930 ± 0.208	<.637
*Rank*	1.000 ± 0.170	0.967 ± 0.186	<.774
*Ctsk*	1.000 ± 0.343	0.933 ± 0.227	<.537
*Acp5*	1.000 ± 0.107	0.933 ± 0.153	<.445

Abbreviations: *Bglap*, osteocalcin; *Calcr*, calcitonin receptor; *Cyp24a1*, 24-hydroxylase; *Sost*, sclerostin.

**Figure 4:**
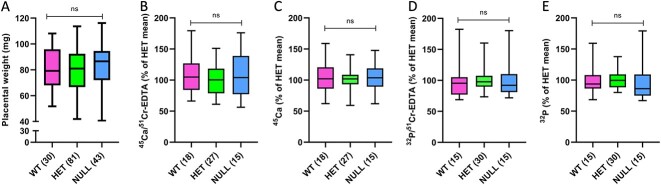
Placental weights and placental transport of calcium and phosphorus. The 24-hydroxylase null fetuses have normal placental weights (A), placental transport of ^45^Ca corrected for ^51^Cr-EDTA diffusion (B), placental transport of ^45^Ca analyzed alone (C), placental transport of ^32^P corrected for ^51^Cr-EDTA diffusion (D), and placental transport of ^32^P analyzed alone (E). The numbers of observations are indicated in parentheses.

Placental weight did not differ among the genotypes (Figure 4A). Placental calcium transport also did not differ between *Cyp24a1* null and WT littermates, whether corrected for ^51^Cr-EDTA or analyzed as ^45^Ca activity alone (Figure 4B and C). Similarly, placental phosphorus transport was also no different among WT and *Cyp24a1* null littermates (Figure 4D and E).

We next examined the expression of genes relevant to calcium and phosphate handling in the placenta (Table 2). The *Cyp24a1* null placentas confirmed the absence of *Cyp24a1* and displayed an increased expression of several genes involved in calcium transport, including *S100g*, *Ncx1*, and *Casr*. Expressions of *Trpv6* and *Cyp27b1* showed numerical increases that did not reach statistical significance. Of phosphate-specific transporters, *NaPi2c* showed significantly decreased expression, while *NaPi2a* and *NaPi2b* were unchanged. *Pth *has a low level of expression in murine placentas,^39^ and it showed a numerical increase in *Cyp24a1* null placentas that did not reach statistical significance. Overall, the findings indicate an altered expression of several calcium-relevant genes and only *NaPi2c* of the phosphate-specific genes in *Cyp24a1* null placentas.

**Table 2:** Relative expression of genes from WT and *Cyp24a1* null placentas. Parameters with statistically significant differences between the two genotypes are shown in bold text. All values have been normalized to the WT; sample sizes were 5 per group.

**Gene**	**WT**	***Cyp24a1* null**	***P*-value**
***Cyp24a1***	**1.000 ± 1.350**	**0.000 ± 0.001**	**<.013**
*Cyp27b1*	1.000 ± 0.377	1.617 ± 0.755	<.141
*Pthrp (Pthlh)*	1.000 ± 0.748	0.715 ± 0.421	<.479
***S100g***	**1.000 ± 0.315**	**2.353 ± 0.691**	**<.006**
*Pmca1*	1.000 ± 0.139	0.909 ± 0.357	<.304
*Trpv6*	1.000 ± 0.209	1.539 ± 0.711	<.142
*Pth*	1.000 ± 0.457	4.376 ± 4.441	<.065
*Vdr*	1.000 ± 0.758	0.634 ± 0.336	<.176
***Ncx1***	**1.000 ± 0.437**	**1.758 ± 0.541**	**<.026**
***Casr***	**1.000 ± 0.433**	**1.927 ± 0.592**	**<.022**
*Pdia3*	1.000 ± 0.118	0.765 ± 0.326	<.168
*Napi2a*	1.000 ± 0.205	0.903 ± 0.296	<.564
*Napi2b*	1.000 ± 0.141	1.055 ± 0.119	<.999
***Napi2c***	**1.000 ± 0.280**	**0.535 ± 0.078**	**<.008**
*Pit1*	1.000 ± 0.538	1.012 ± 0.210	<.963
*Pit2*	1.000 ± 0.377	1.190 ± 0.285	<.395
*Xpr1*	1.000 ± 0.472	1.091 ± 0.089	<.683

Abbreviation: *Cyp24a1*, 24-hydroxylase.

**Figure 5:**
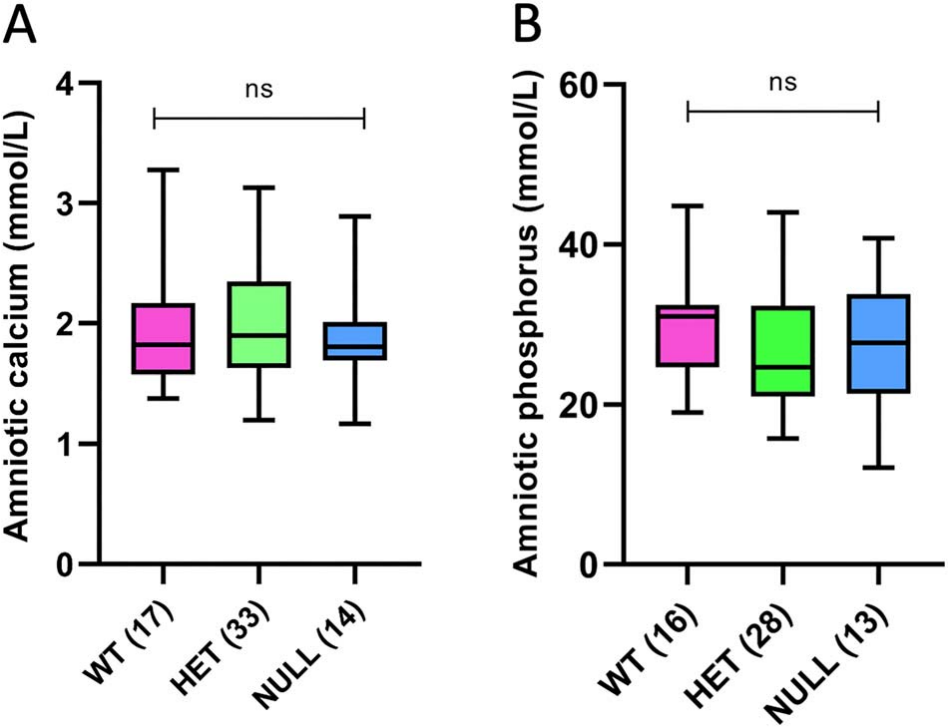
Amniotic fluid mineral concentrations. Amniotic fluid largely consists of urine and its mineral content reflects renal mineral excretion. The concentrations of calcium (A) and phosphorus (B) were no different in 24-hydroxylase null fetuses compared to their littermates. The numbers of observations are indicated in parentheses.

Finally, we examined the aspects of fetal renal function. Amniotic fluid, which largely consists of urine, showed no differences in the calcium or phosphorus concentrations among the fetal genotypes (Figure 5A and B). With respect to renal gene expression, *Cyp24a1* null fetal kidneys showed the absence of *Cyp24a1*, significantly reduced expression of *Cyp27b1,* and near-significant increased expression of *S100g*. The remaining calciotropic and phosphotropic genes showed no significant alterations in expression (Table 3).

**Table 3:** Relative expression of genes from WT and *Cyp24a1* null kidneys. Parameters with statistically significant differences between the two genotypes are shown in bold text. All values have been normalized to the WT; sample sizes were 5 per group.

**Gene**	**WT**	***Cyp24a1* null**	***P*-value**
***Cyp24a1***	**1.000 ± 0.538**	**0.000 ± 0.001**	**<.003**
***Cyp27b1***	**1.000 ± 0.638**	**0.149 ± 0.115**	**<.019**
*S100g*	1.000 ± 0.261	1.335 ± 0.272	<.082
*Pmca1*	1.000 ± 0.206	1.025 ± 0.085	<.812
*Trpv6*	1.000 ± 0.226	0.940 ± 0.254	<.703
*Vdr*	1.000 ± 0.304	1.225 ± 0.332	<.296
*Ncx1*	1.000 ± 0.372	1.004 ± 0.121	<.994
*Casr*	1.000 ± 0.260	1.080 ± 0.291	<.605
*Napi2a*	1.000 ± 0.305	0.906 ± 0.266	<.616
*Napi2b*	1.000 ± 0.141	1.385 ± 0.640	<.225
*Napi2c*	1.000 ± 0.358	0.838 ± 0.435	<.529

Abbreviation: *Cyp24a1*, 24-hydroxylase.

## Discussion

In this study, we found that ablation of *Cyp24a1* increased fetal calcitriol, which was accompanied by hypercalcemia, modest hypophosphatemia, increased FGF23, and normal skeletal development and mineralization. Serum P1NP was reduced as was the expression of the osteoblast genes *Sost* and *Bglap*, while serum CTX was normal and an increase in *Calcr* was the only change among osteoclast-relevant genes. Placental calcium and phosphate transports were normal, although several genes involved in the calcium transport were upregulated, while all phosphate-relevant genes were unchanged except for *NaPi2c*, which has a low expression in placenta compared to *NaPi2b*.

The development of fetal hypercalcemia confirms that the phenotype of *Cyp24a1* ablation begins in utero. The hypercalcemia may be caused by calcitriol-mediated increased expression of genes involved in placental calcium transport, which cause the net calcium transport to be inappropriately normal rather than reduced in compensation to the fetal hypercalcemia. In support of this, we have previously found that fetal hypercalcemia was accompanied by a reduced placental calcium transport.^42^ The lack of any change in the skeletal mineral content or renal calcium excretion into amniotic fluid is consistent with no marked differences in the net delivery of calcium from the placenta. However, the reduction in P1NP, the reduced expressions of *Sost* and *Bglap*, and the increased expression of *Calcr* allow for the possibility that a small reduction in the influx of calcium into bone (via osteoblasts) or efflux of calcium out of bone (via osteoclasts) contributed to the hypercalcemia without a detectable change in the skeletal mineral content. The mineral concentrations in the fetal circulation represent <1% of the mineral content of the skeleton; therefore, a small change in the former can occur without necessitating a change in the latter. We cannot rule out a small increase in placental calcium transport that could not be resolved with the assay, or that the set-point for serum calcium was higher in *Cyp24a1* null fetuses compared to their littermates. During fetal development, the intestines are not an important route of mineral delivery for the fetus (only amniotic fluid is available to absorb), and the intestines respond poorly to calcitriol until after birth^2^; thus, alterations in intestinal calcium absorption are unlikely to occur or to be of physiological significance.

The *Cyp24a1* null fetuses are modestly hypophosphatemic compared to their heterozygous littermates. This contrasts to the hyperphosphatemia that develops postnatally, likely from an increased intestinal phosphate absorption. We did not find a lower placental phosphate transport, but a small decrease cannot be excluded. Moreover, the assay measures a predominantly forward flow of phosphate from the maternal to fetal circulation over 5 min. If higher calcitriol increases the reverse flow of phosphate from the fetal to maternal circulation, this would not be detected due to the forward-transported isotope being diluted within the fetal circulation before any might return to the maternal circulation. The 3.5-fold increase in FGF23 conceivably reduces serum phosphorus by increasing the renal phosphorus excretion or affecting placental phosphate transport. However, we did not find any difference in the phosphorus content of amniotic fluid or in placental transport. Moreover, in prior work, we found that a 7.8-fold increase in FGF23 in *Hyp* mice (a model of X-linked hypophosphatemic rickets in humans) did not alter the fetal serum phosphorus, amniotic fluid phosphorus, or placental phosphate transport.^43^ Similarly, ablation of the genes encoding FGF23 or its co-receptor Klotho did not disturb fetal serum phosphorus, amniotic fluid phosphorus, or placental phosphate transport.^43^^,^^44^ It is only when maternal hyperphosphatemia was induced through dietary phosphate loading that a role for FGF23 to protect against fetal hyperphosphatemia was detected.^45^

The potential effect of increased calcitriol on fetal mineral homeostasis has previously been studied by exogenously administering active vitamin D metabolites to the mother or fetus. Treatment of the mother with calcitriol or 1α-hydroxyvitamin D (1α-OHD) increased placental calcium and phosphate transport in fetal lambs and guinea pigs,^46^^,^^47^ while the administration of calcitriol to the fetal side of in situ perfused placentas from nephrectomized fetal lambs also increased placental calcium transport.^48^ With respect to the effects on fetal serum mineral concentrations, active vitamin D metabolites given to the mother or fetus directly had a consistent effect to increase the fetal serum calcium,^46–49^ whereas the effects on serum phosphorus were variable. When injected directly into fetal rats, calcitriol decreased serum phosphorus.^49^ When calcitriol was administered to pregnant guinea pigs, it reduced fetal serum phosphorus at mid-gestation, but it increased serum phosphorus by late gestation.^47^ When 1α-OHD was given to pregnant sheep, it caused increased serum phosphorus in fetal lambs at mid-gestation but no change in serum phosphorus at late gestation.^46^ Overall, there is a precedent for increased calcitriol not only to increase fetal serum calcium but also to reduce fetal serum phosphorus. The effects of pharmacological administration of calcitriol or 1α-OHD are not necessarily the same as endogenous increases in fetal calcitriol, such as occurred in *Cyp24a1* null fetuses. Moreover, the administration of active vitamin D metabolites to the dam can potentially alter placental mineral transport by acting on both the maternal and fetal sides of the placenta. Conversely, in the current mouse model, calcitriol was increased in the *Cyp24a1* null fetuses but not in their mothers.

The initial report of the *Cyp24a1* null phenotype examined the offspring of *Cyp24a1* null dams at 5 d after birth, and it was found that *Cyp24a1* null pups had an impaired mineralization of intramembranous bone, whereas their *Cyp24a1^+/−^* littermates did not.^27^ The authors concluded that calcitriol was higher in *Cyp24a1* null fetuses, likely due to the combined effects of increased maternal and fetal production of calcitriol, and speculated that high levels of calcitriol were directly causing the impaired mineralization. They generated double-mutants that lacked both *Cyp24a1* and *Vdr*, which rescued the bone phenotype, thereby confirming that it was mediated by calcitriol acting on the VDR.^27^ We found no bone phenotype at ED 18.5 despite high levels of calcitriol in *Cyp24a1* null fetuses as compared to WT. Two reasons contribute to this. First, serum calcitriol should not be increased in *Cyp24a1^+/−^* dams as much as in *Cyp24a1* null dams. Second, our studies were done the day prior to birth and not 5 d after birth, which is when the intramembranous bone phenotype was first identified. The studies in the present manuscript were designed so that the phenotype of *Cyp24a1* null fetuses, as compared to their WT and *Cyp24a1^+/−^* littermates, must result only from the loss of the fetal gene; each fetus experiences an identical intrauterine environment that includes the normal maternal serum calcium and phosphorus. Conversely, *Cyp24a1* null fetuses of *Cyp24a1* null mothers will have a phenotype resulting from a combination of several factors: loss of both copies of the maternal gene causing an altered maternal milieu (high calcitriol, FGF23, and calcium); fetal compensatory responses to the altered maternal mineral and hormone levels; and loss of both copies of the fetal gene leading to altered physiology in the *Cyp24a1* null fetus itself. Study of such *Cyp24a1* null fetuses from Cyp24a1 null dams is beyond the scope of the current work, but this is planned for the future.

There may be differences between the murine and human phenotype caused by the loss of CYP24A1. However, in the absence of any human data, these findings are relevant to humans by predicting that infantile hypercalcemia type 1 onsets in utero such that affected human babies should have increased cord blood calcium, reduced serum phosphorus, and increased FGF23, but no skeletal abnormality at birth. The exposure to high levels of serum calcium from early in gestation may also predispose affected children or adults to develop hypocalcemic symptoms if the serum calcium is reduced into the normal range that they are unaccustomed to. This is analogous to patients with inactivating mutations of the calcium sensing receptor, for whom life-long hypercalcemia also onsets in utero (as suggested by the animal model); they are unaccustomed to a “normal” serum calcium.^42^^,^^50^

In conclusion, loss of *Cyp24a1* in fetal mice causes hypercalcemia, modest hypophosphatemia, and increased calcitriol and FGF23, but no alteration in skeletal development, and it predicts that human fetuses bearing homozygous or compound heterozygous inactivating mutations of *CYP24A1* will also be hypercalcemic in utero but with normal skeletal development.

## Supplementary Material

Supplementary_Figure_1_ziae012

Legend_for_Suppl_Figure_1_plus_Suppl_Table_1_ziae012

## Data Availability

Data are available on request from the authors.
