# Supplementary figures and images for: Loss of 24-hydroxylated catabolism increases calcitriol and fibroblast growth factor 23 and alters calcium and phosphate metabolism in fetal mice
Source: JBMR Plus. 2024 Jan 29;8(5):ziae012. doi: 10.1093/jbmrpl/ziae012 (PMC10993470; doi:10.1093/jbmrpl/ziae012)

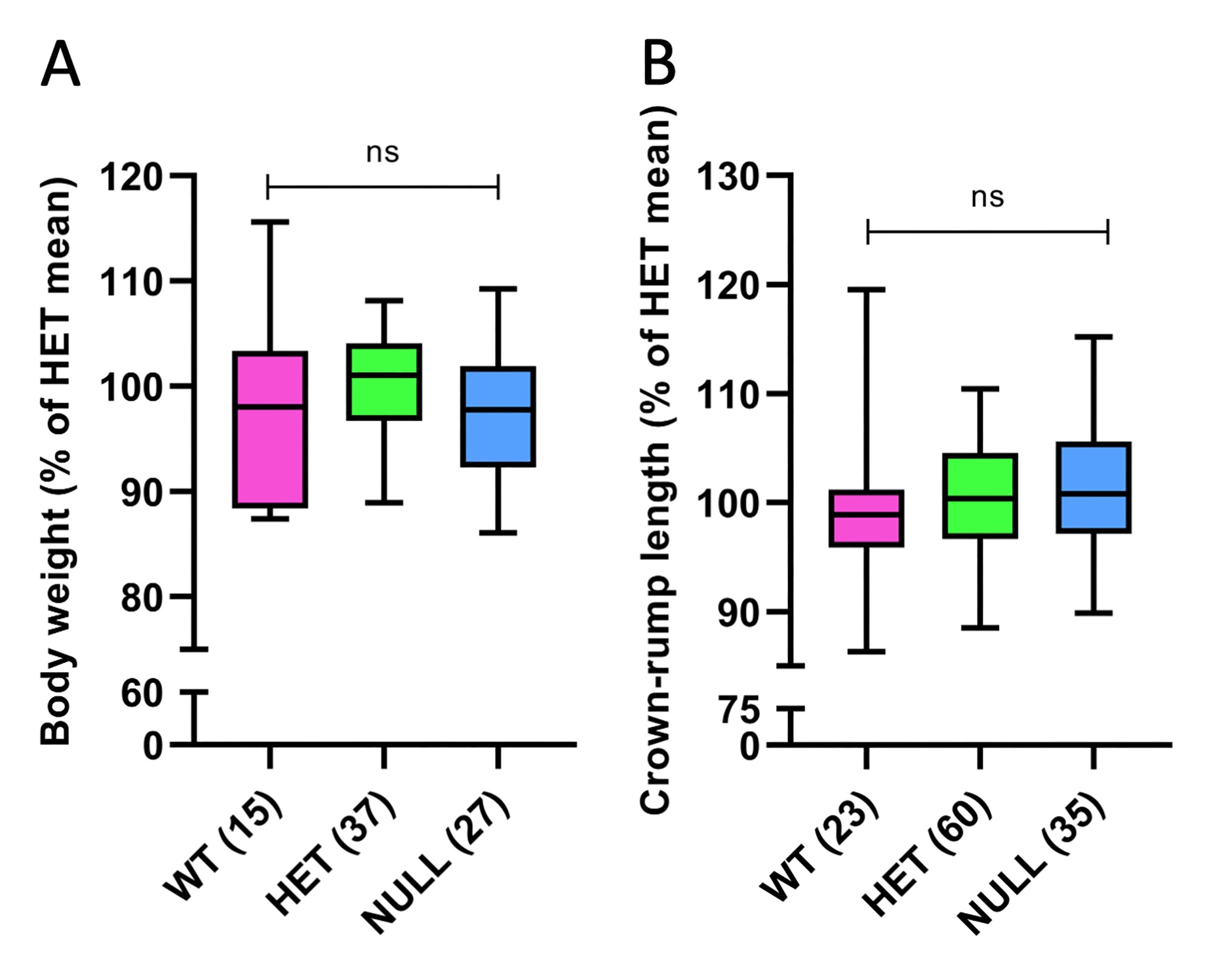

Supplement: Supplementary_Figure_1_ziae012 [file supplementary_figure_1_ziae012.jpeg]
