# Supplementary material for: Loss of 24-hydroxylated catabolism increases calcitriol and fibroblast growth factor 23 and alters calcium and phosphate metabolism in fetal mice
Source: JBMR Plus. 2024 Jan 29;8(5):ziae012. doi: 10.1093/jbmrpl/ziae012 (PMC10993470; doi:10.1093/jbmrpl/ziae012)
Supplement: Legend_for_Suppl_Figure_1_plus_Suppl_Table_1_ziae012 [file legend_for_suppl_figure_1_plus_suppl_table_1_ziae012.docx]

**Supplementary Figure 1**. Body weight and crown-rump lengths adjusted for litter sizes. To control for variability in fetal size from litter to litter, results were normalized to the mean values of *Cyp24a1^+/-^* pups within each litter. There were no differences in adjusted body weight (A) or crown-rump length (B). The numbers of observations are indicated in parentheses.

**Supplementary Table 1.** Primer sequences for qPCR. All TaqMan primers obtained from Thermo Fisher Scientific, Canada.

| Gene name | Catalogue / Assay ID |
| --- | --- |
| *Cyp24a1* | Mm00487245_m1 |
| *Pdia3* | Mm00433130_m1 |
| *Casr* | Mm00443375_m1 |
| *Pmca1* | Mm01245805_m1 |
| *Ncx1* | Mm01232254_m1 |
| *Vdr* | Mm00437297_m1 |
| *Pth* | Mm00451600_g1 |
| *S100g* | Mm00486654_ml |
| *Cyp27b1* | Mm01165922_g1 |
| *Pthrp (Pthlh)* | Mm00436057_m1 |
| *Napi2a* | Mm00441450_m1 |
| *Napi2b* | Mm00448749_m1 |
| *Napi2c* | Mm00551746_m1 |
| *Trpv6* | Mm00499069_m1 |
| *Pit1* | Mm00489378_m1 |
| *Pit2* | Mm00660203_m1 |
| *Xpr1* | Mm00495501_m1 |
| *Runx2* | Mm00501584_m1 |
| *Sost* | Mm00470479_m1 |
| *Alp1* | Mm00475834_m1 |
| *Ibsp* | Mm00492555_m1 |
| *Bglap* | Mm03413826_m1 |
| *Pthr1* | Mm00441046_m1 |
| *Rank* | Mm00437132_m1 |
| *Ctsk* | Mm00484093_m1 |
| *Acp5* | Mm00475698_m1 |
| *Calcr* | Mm00432282_m1 |
| *Gapdh* | Mm99999915_g1 |
